# Changes in the Percentages of B- and T-Lymphocytes and Antibody Titres in Laying Hens Infested with *Dermanyssus gallinae*—A Preliminary Study

**DOI:** 10.3390/ani10060987

**Published:** 2020-06-05

**Authors:** Sylwia Koziatek-Sadłowska, Rajmund Sokół

**Affiliations:** Department of Parasitology and Invasive Diseases, Faculty of Veterinary Medicine, University of Warmia and Mazury in Olsztyn, ul. Oczapowskiego 13, 10-719 Olsztyn, Poland; sylwia.koziatek@uwm.edu.pl

**Keywords:** *D. gallinae*, hematophagous ectoparasite, poultry red mite, antibody titre, lymphocyte subpopulation

## Abstract

**Simple Summary:**

*Dermanyssus gallinae,* a hematophagous ectoparasite, adversely affects the health status of laying hens, leading to reduced egg production and significant economic losses in commercial poultry farms. The aim of this study was to determine the effect of *D. gallinae* on the immunological parameters (three lymphocyte subpopulations) and post-vaccination antibody titres (against most important avian pathogens) in layer hens during the egg production cycle. A total of 80 blood samples were collected at four time-points (B1–B4) from 10 Hy-Line Brown hens naturally infested with *D. gallinae*, which were randomly selected from a commercial layer farm. The infestation was monitored and treated twice with Biobeck PA 910 (active ingredient AI – silicon dioxide). The samples were collected before and after each treatment. The percentages of subpopulations of B cells and helper (Th) and cytotoxic (Tc) T cells were determined by flow cytometry. Antibody titres were determined by the immunoenzymatic method. The percentage of Th cells and post-vaccination anti-infectious bronchitis virus (anti-IBV) and anti-Newcastle disease virus (anti-NDV) antibodies decreased significantly at the second infestation peak when the number of parasites was twice higher than at the first infestation peak. There were non-significant correlations between the number of mites and antibody titres. These findings suggested that *D. gallinae* might inhibit humoral immune responses since the percentages of B cells and Th cells were negatively correlated with the number of mites. The percentage of Tc cells was positively correlated with the number of mites, which indicated that *D. gallinae* could stimulate cellular immune responses in infested laying hens. However, further research is needed to determine whether *D. gallinae* suppresses the production of vaccine-induced antibodies.

**Abstract:**

(1) Background: *Dermanyssus gallinae*, a hematophagous ectoparasite, adversely affects the health status of laying hens, leading to reduced egg production and significant economic losses in commercial farms. The aim of this study was to determine the effect of *D. gallinae* on the development of post-vaccination immune responses in layer hens. (2) Methods: A total of 80 blood samples were collected at four time-points (B1–B4) from 10 Hy-Line Brown hens, randomly selected from a commercial layer farm. The flock was naturally infested with *D. gallinae* and treated twice with Biobeck PA 910 (AI silicon dioxide). The samples were collected before and after each treatment. The percentages of IgM+ B cells, CD3+/CD4+ T cells and CD3+/CD8a+ T cells were determined by flow cytometry; the titres of antibodies against avian encephalomyelitis, infectious bronchitis virus, Newcastle disease virus, *Ornithobacterium rhinotracheale*, reticuloendotheliosis virus and avian reovirus were determined by the immunoenzymatic method. (3) Results: The percentage of Th cells and post-vaccination anti-IBV and anti-NDV antibodies decreased significantly at the second infestation peak when the number of parasites was twice higher than at the first infestation peak. Non-significant negative correlations were found between the number of mites and the percentage of B cells (R = −0.845, *p* > 0.05) and between the number of mites and the percentage of Th cells (R = −0.522, *p* > 0.05), and a significant positive correlation was noted between the number of mites and the percentage of Tc cells (R = −0.982, *p* < 0.05). There were non-significant correlations between the number of mites and antibody titres. (4) Conclusion: The present findings suggested that *D. gallinae* might inhibit immune responses since the percentages of B cells and Th cells were negatively correlated with the number of mites. The percentage of Tc cells was positively correlated with the number of mites, which indicated that *D. gallinae* could stimulate cellular immune responses in infested laying hens. However, further research is needed to determine whether *D. gallinae* suppresses the production of vaccine-induced antibodies.

## 1. Introduction

*Dermanyssus gallinae* (De Geer, 1778) (*D. gallinae*), commonly known as poultry red mite (prm), is a temporary ectoparasite, feeding on the blood of wild and domestic birds [1,2,3,4]. It is currently the most serious mite pest in commercial caged layer hen farms [5,6,7,8,9]. Farm conditions favour mass occurrence and uncontrolled growth of the parasite. Such conditions include high, constant temperature, high relative air humidity, constant access to the host body and long production cycle (80 weeks) [8,10,11]. The parasite settles in numerous gaps in the poultry house construction and equipment where it goes through the development cycle of five life stages (egg, larva, protonymph, deutonymph and adults). It needs the host’s blood for growth in the last three stages [1,12]. They feed briefly and return to their refugia [13,14]. This way of life makes it poorly accessible to acaricides [8,10,15]. Moreover, there have been increasing numbers of reports on the prm resistance to acaricides [16,17,18]. The implementation of enriched cages (in line with the EU Directive 1999.74/EC) has probably led to a significant and uncontrolled proliferation of *D. gallinae* [8,19]. Under heavy infestation, the welfare of the laying hens significantly decreases. Upon puncturing the skin of the host, the mite introduces toxic saliva that can cause itching and irritation, which leads to distress and, consequently, poor feed conversion, reduced egg production and increased bird mortality [10,20,21].

Birds in commercial flocks of laying hens are given preventive vaccination until the 20th week of their lives. Not only are they important from the economic perspective, but they ensure consumer safety. Vaccination effectiveness may be affected by feed, genetic, environmental, chemical and biological (bacteria and viruses) factors. According to the literature, hens, which are chronically exposed to *D. gallinae*, do not produce a strong immune response against this parasite. A non-significant correlation between serum IgY level and red mite population levels has been observed [22]. Another study has revealed that *D. gallinae* feeding stimulates Th1 and pro-inflammatory cytokines/chemokines, initially followed by their subsequent down-regulation [23]. There is also a theory that *D. gallinae* might adopt a feeding strategy of minimal host interference, while *D. gallinae* could determine host immune status by nymphal/larval survival rates [24]. Our recent studies revealed that *D. gallinae* infestation caused somatic and psychogenic stress in layer hens, which led to lower humoral immunity decrease of γ-globulin in the blood of hens infested by *D. gallinae* [25,26]. Therefore, the question may be formulated, whether the pressure (feeding) of *D. gallinae* may have an immunosuppressive effect and affect the development of post-vaccination immune responses in laying hens. There has been no scientific research on the subject. The research on *D. gallinae*, in an ectoparasite–host immunological relationship, mainly concerns vaccines against this parasite.

Therefore, the aim of this study was to determine the percentages of subpopulations of B IgM+ cells, T cells CD3+/CD4+ (Th) and T cells CD3+/CD8a+ (Tc) by flow cytometry and the titres of antibodies against AE (avian encephalomyelitis), IBV (infectious bronchitis virus), NDV (Newcastle disease virus), ORT (*Ornithobacterium rhinotracheale*), REV (reticuloendotheliosis virus) and REO (avian reovirus) using the immunoenzymatic method with ELISA tests (IDEXX, USA) in the peripheral blood of laying hens infested with *D. gallinae*, controlled with Biobeck PA 910 (active ingredient (AI) silicon dioxide).

## 2. Materials and Methods

### 2.1. Birds

Blood samples for analyses were collected from 10 randomly selected laying hens from an industrial laying hen farm naturally infested with *D. gallinae*. A flock of 56,400 hens of the Hy-Line Brown line was kept in battery cages for 54 weeks in conditions complying with the valid standards. Hens were fed with high-quality feed and watered *ad libitum* from automatic drinkers. The air temperature in the hen house was 20–22 °C, and the relative humidity (RH) was 40%–60%. The birds were given prophylactic vaccinations as per the standard programme: *Salmonella enteritidis*—6–10 days, and 70–74 days*, Staphylococcus gallinarum—*42–46 days and 16 weeks,* Mycoplasma gallisepticum*, *Mycoplasma synoviae—*56–60 days, infectious bronchitis virus (IBV) and Newcastle disease virus (NDV)*—*35–39 days and 13 weeks, Gumboro disease (IBD)—22–26 days and 30–34 days, avian encephalomyelitis (AE) and egg drop syndrome (EDS’76)—12–13 weeks. Day or week indicates the age range of the hen while vaccinated.

All procedures were conducted according to the prevailing national legislation on the use of animals in research. The experiment was carried out in compliance with the recommendations of the Local Ethics Committee for Animal Experimentation in Olsztyn (no. 59/2006).

### 2.2. Monitoring of the Dermanyssus gallinae Infestation

**Figure 1:**
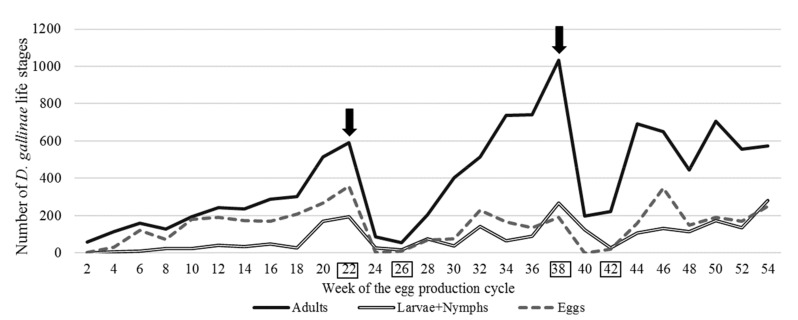
The size of the *Dermanyssus gallinae* population and its life stages following two applications of the acaricide product Biobeck PA 910 (AI silicon dioxide, Biologische Preparate, Brilon, Germany) during a 54-week production cycle. (Mite treatment MTI and MTII dates are marked by arrows, blood sampling B1–4 dates are marked by squares).

The infestation of *D. gallinae* was monitored in 2-week intervals. The parasites were caught with a system of traps fixed to the cages under the conveyor belt for egg collection, four at each of fixed selected places, on the second and the fourth row of cages [27]. The traps from each site were collected separately to a twist-type jar (0.9 L) and sent to the laboratory. The jars with their contents were cooled down for 30 min at a temperature of −20 °C to immobilise the parasites and count them. The deposit was poured onto a 2 × 2 cm chequered Petri dish, and the parasite life stages; (a) adults, (b) larvae + nymphs, (c) eggs were counted with a binocular magnifying glass (Olympus, Type SZ, magn. 40×, Tokyo, Japan). The infestation size was presented as the average number of each life stage (Figure 1). 

### 2.3. Controlling of the Dermanyssus gallinae Infestation

The infestation of *D. gallinae* was controlled with Biobeck PA 910 (AI silicon dioxide, Biologische Preparate, Brilon, Germany), which was applied in accordance with the manufacturer’s guidelines at 1–2 g/hen by spraying onto the cages and hens with a device imparting static electricity to it. Two dates for mite treatment were established based on the results of ongoing monitoring of the number of different life stages. It was performed in the 22nd and 38th week of the production cycle. The first mite treatment (MTI) was performed after a rapid increase in the number of *D. gallinae* was observed—a nearly two-fold increase in the number of adults and eggs and a four-fold increase in the number of larvae + nymphs compared to week 16 (Figure 1). The second mite treatment (MTII) was performed after a two-fold increase, in the number of adults and larvae + nymphs, was recorded compared to week 32 (the number of eggs was similar).

### 2.4. Collection of the Blood Samples

Blood samples for cytometric and serological tests were collected at four time-points during the egg production cycle, from the wing vein of 10 randomly selected hens for each analysis. Blood samples were collected in the 22nd—B1 (before MTI), 26th—B2 (after MTI), 38th—B3 (before MTII), 42nd—B4 (after MTII) week of the production cycle. Hens were 40, 44, 56, 60 weeks old, respectively, in B1–B4. A total of 80 blood samples were tested (40 for each analysis). Hens were marked, and the blood was taken from the same hens each time.

### 2.5. Flow Cytometry Analysis

Blood samples in the amount of 1.5 mL were collected into test tubes with an anticoagulant EDTA-K2 (Sarstedt). Subsequently, blood was diluted 1:1 with PBS (phosphate-buffered saline) with 1% fetal calf serum (FCS). The blood samples were layered on 3 mL of Histopaque-1077 gradient (Sigma-Aldrich, Darmstadt, Germany) and centrifuged in 15 mL FALCON tubes at 400× g at the temperature of 25 °C for 30 min. After centrifuging, the layer of mononuclear cells was carefully collected into sterile tubes and washed twice with PBS with an addition of 1% FCS and subsequently suspended in 1 mL PBS. The cells were then counted by the chamber method. A total of 10^6^ cells were collected from the suspension and transferred to cytometric tubes. Two microlitres of antibodies targeted at surface domains of the CD3 receptor (Mouse AntiChicken CD3-FITC clone CT-3, SouthernBiotech, Birmingham, AL, USA), CD4 receptor (Mouse AntiChicken CD4-FITC clone CT-4, SouthernBiotech, Birmingham, AL, USA) and CD8 receptor (Mouse AntiChicken CD8a-Cy5 clone 3-298, SouthernBiotech, Birmingham, AL, USA), T cells and immunoglobulin BCR (IgM^+^) of B cells (Mouse AntiChickenIgM-SPRD clone M-1, SouthernBiotech, Birmingham, AL, USA) were added to each tube. The samples were incubated on ice for 30 min in darkness. Subsequently, the cells were washed twice in PBS, centrifuged at 250× *g* for 7 min at 4 °C, and the resulting cell pellets were suspended in 0.5 mL of fixing buffer (CellFIX, Becton Dickinson, Franklin Lakes, NJ, USA) and refrigerated. The samples were tested after 18 h with a flow cytometer FACSCanto II (Becton Dickinson, Franklin Lakes, NJ, USA). Immunophenotypic analysis of the lymphocytes was performed with a specialist software FlowJo (Tree Star, Inc., Ashland, OR, USA).

### 2.6. Serological Analysis (ELISA)

Blood samples were taken to sterile tubes and refrigerated for 1 h to separate the serum from morphotic elements. The blood was then centrifuged for 20 min at 4 °C at 1000× g. Portions of 1.5 mL of serum were transferred to microcentrifuge tubes. The titres of antibodies against AE (avian encephalomyelitis), IBV (infectious bronchitis virus), NDV (Newcastle disease virus), ORT (*Ornithobacterium rhinotracheale*), REO (avian reovirus) and REV (reticuloendotheliosis virus) were determined in accordance with the manufacturer’s guidelines for commercial tests ELISA Pro Flok HEV Ab (Zoetis, Parsippany, NJ, USA). The absorbance was read out with a BioTek ELx800 (Winooski, VT, USA) plate reader. The data analysis and calculations were performed in the xCheck IDEXX (Westbrook, ME, USA) software environment.

### 2.7. Statistical Analysis

The results were analysed statistically by calculating mean values, standard error (SE) and coefficient of variation (CV%) for antibody titres. Differences between B1–B4 for lymphocyte subpopulations and antibody titres were analysed using repeated-measures ANOVA test or MANOVA test if the assumption of sphericity was violated (statistically significant Mauchly test). Tukey’s post hoc test was used. The statistical significance was declared at *p* < 0.05. Pearson correlation coefficient test was used to determine the relationships between mite numbers and lymphocyte subpopulations and antibody titres.

## 3. Results

### 3.1. Development of the Dermanyssus gallinae Infestation

The mean number of different life stages of *D. gallinae* (adults, larvae + nymphs and eggs) in consecutive weeks of the production cycle of laying hens; dates of mite treatment (MTI and MTII); dates of blood sampling for assays (B1–B4) are shown in Figure 1. Two peaks in mite population (PI and PII, in the study called ‘infestation peak’) during the 54-week production cycle induced by mite treatment were recorded. The growth of the *D. gallinae* population was moderate until week 18. Subsequently, the growth rate increased. A two-fold increase in the number of adults, larvae + nymphs and eggs of *D. gallinae* was recorded in week 22 of the production cycle (592 adults, 194 larvae + nymphs and 357 eggs) compared to week 18. Mite treatment I (MTI) was performed, as a result of which the number of life stages of *D. gallinae* was reduced significantly (found: 56 adult forms, 16 larvae + nymphs and 10 eggs) in week 24 of the production cycle compared to week 22. The infestation remained at a low level for 2 weeks. Subsequently, it started to grow rapidly, starting with week 26. The reinfestation peak (PII) was observed in week 38 of the production cycle (found: 1034 adult forms, 268 larvae + nymphs and 191 eggs). The second mite treatment (MTII) was performed, which resulted in a statistically significant reduction in the number of individual life stages of *D. gallinae* in week 40 of the production cycle (found: 199 females, 124 larvae-nymphs and 1 egg). The effect persisted for the next 2 weeks. After that, the population started to regenerate, but statistically non-significant fluctuations of the numbers of individual life stages were observed. Adults in a number of 574, 282 larvae-nymphs and 251 eggs were observed in the 54th (final) week.

Each mite treatment procedure resulted in a reduction in the number of individual life stages for 2 weeks. After that time, the population re-grew, and the infestation development rate after the second mite treatment procedure was 2.4 times higher than the infestation development rate compared to MTI.

### 3.2. Changes in the Percentages of Lymphocytes Subpopulations

**Figure 2:**
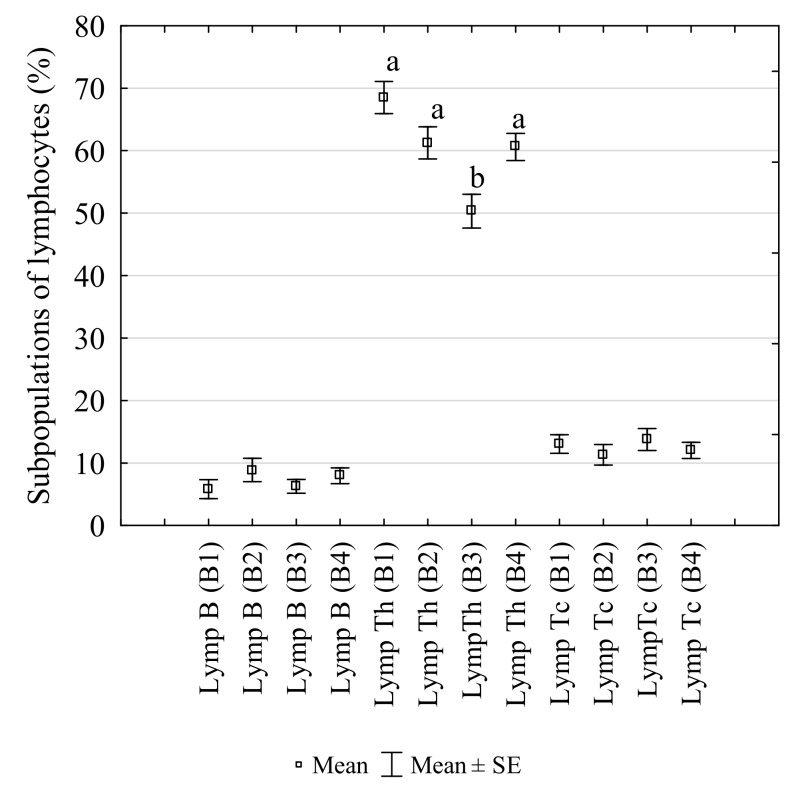
Changes in the percentages of B IgM+ cells (Lymp B), T CD3+CD4+ cells (Lymp Th) and T CD3+CD8a+ cells (Lymp Tc) in the peripheral blood of laying hens infested with *Dermanyssus gallinae*, controlled with Biobeck PA 910 (AI silicon dioxide, Biologische Preparate, Brilon, Germany). (B1–B4: blood sampling dates; a,b: different letters indicate statistical differences between groups).

The percentages and changes in the populations of Th cells, Tc cells and B cells in laying hens infested with *D. gallinae* controlled with Biobeck PA 910 (AI silicon dioxide, Biologische Preparate, Brilon, Germany) are shown in Figure 2. The Th cells accounted for the highest portion of all the lymphocytes, followed by B cells and Tc cells in each assay (B1–4). There were 68.52% of Th cells of total lymphocytes at the infestation peak I (B1). The *D. gallinae* population decreased after Biobeck PA 910 (AI silicon dioxide, Biologische Preparate, Brilon, Germany) was applied (B2), and the percentage of the lymphocytes decreased non-significantly (61.25%), and the decrease was continued significantly at the infestation peak II (B3) (50.34%) compared to B1 (*p* = 0.0002) and B2 (*p* = 0.018). Subsequently, the percentage increased to 60.6% (*p* = 0.028) after another mite treatment compared to B3. The analysis of correlation revealed a non-significant negative correlation between mite number and B cells (R = −0.845, *p* > 0.05), a non-significant negative correlation between mite number and Th cells (R = −0.522, *p* > 0.05) and a significant positive correlation between mite number and Tc cells (R = −0.982, *p* < 0.05).

### 3.3. The Changes in the Titres of Antibodies

**Figure 3:**
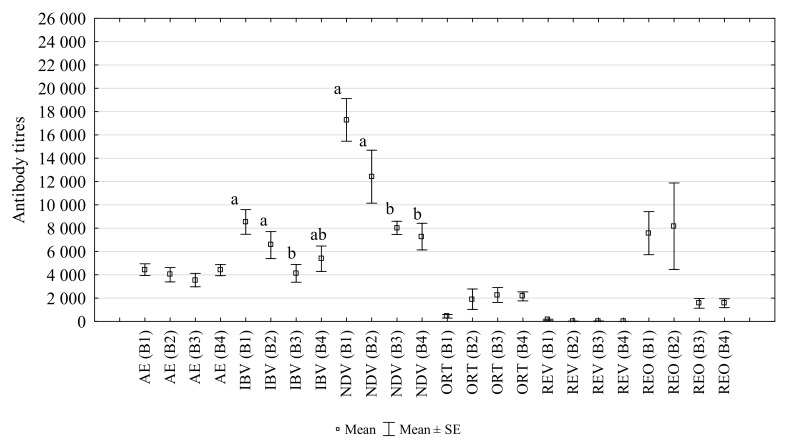
Changes in the titres of antibodies against AE (avian encephalomyelitis), IBV (infectious bronchitis virus), NDV (Newcastle disease virus), ORT (*Ornithobacterium rhinotracheale*), REV (reticuloendotheliosis virus) and REO (avian reovirus) in the peripheral blood of laying hens infested with *D. gallinae*, controlled with Biobeck. (B1–B4: blood sampling dates; a, ab, b: different letters indicate statistical differences between groups).

The titres of antibodies against AE, IBV, NDV, REV, REO and ORT in laying hens infested with *D. gallinae*, controlled with Biobeck PA 910 (AI silicon dioxide, Biologische Preparate, Brilon, Germany), are shown in Figure 3. The study found a significant decrease in the titre of post-vaccination antibodies against IBV and NDV at the second infestation peak (B3) (*p* = 0.014 and *p* = 0.001, respectively). The titres of post-vaccination antibodies against AE did not change significantly during the study. However, a non-significant negative correlation was observed between mite numbers and AE titre (R = −0.564, *p* > 0.05). Antibodies against REO, REV and ORT were found despite the hens were not immunised earlier against those pathogens. The titres of antibodies against REO in subsequent weeks of the study were 7472, 8163, 2246, 1556. The titres of antibodies against REV were 121, 14, 19, 1. The titres of antibodies against ORT increased during the production cycle and were 402, 1904, 2921, 2150.

## 4. Discussion

This paper was the first report on the effect of *D. gallinae* infestation on a post-vaccination immune response in laying hens during the production cycle. Due to difficulties with access to the hen house free from the infestation of *D. gallinae*, we could not include a control group. Nevertheless, blood samples for cytometric and serological analyses were collected at the infestation peaks (on the day of mite treatment) and after the infestation subsided was recorded. Thus, we could observe the changes in the studied parameters under high and low mite pressure, which showed how *D. gallinae* might affect these parameters.

In the first infestation peak, no significant change in the percentages of B, Th or Tc cells or antibodies was observed. In the re-infestation of *D. gallinae* (infestation peak II), a significant decrease in the Th cells and IBV and NDV post-vaccination antibodies was observed.

Helper lymphocytes (Th) take part in the development of humoral response. They facilitate activation, proliferation and differentiation of B and Tc cells and have a CD4+ receptor on their surface. They also recognise antigens after they bind to class II of major histocompatibility complex (MHC) cells [28,29]. Our study showed a significant decrease in the percentage of these lymphocytes at the infestation peak II and a subsequent increase when the infestation subsided. These findings suggested that *D. gallinae* could inhibit the development of humoral immune response in infested laying hens.

Cytotoxic lymphocytes (Tc) play an important role in acquiring post-vaccine immunity. They are also called antigen-specific since they have a CD8+ receptor on their surface. A CD3+ receptor is present on the surface of most mature T cells and thymocytes. They are capable of destroying cells infected by a virus, cancer cells and of recognising proteins and glycoproteins (antigens) [28]. The percentage of these lymphocytes, as observed in this study, did not change significantly during the experiment. However, a strong positive correlation between mite numbers and those cells was reported. The percentage of these lymphocytes was seen to decrease during the periods of low *D. gallinae* infestation. These findings suggested that the parasite could stimulate the cellular immune response in infested laying hens, but the infestation was not sufficiently high to produce a significant effect.

B cells play a crucial role in the humoral immune response. They differentiate into memory cells and/or plasmatic cells, which produce antibodies specific to protein antigens in a complex process in which antigen-specific CD4+ helper cells take part [29,30]. Like Tc cells, the percentage of these lymphocytes, observed in this study, did not change significantly during the experiment. Only an increasing trend was observed for these lymphocytes during the period of low *D. gallinae* infestation. Despite the absence of statistical significance, these findings might suggest that *D. gallinae* could inhibit the post-vaccination humoral immune response. This was also testified to by a decrease in the post-vaccination antibody titre during the period of high *D. gallinae* infestation (Figure 3).

The titres of post-vaccination antibodies of AE, IBV, NDV changed during the cycle in a similar manner (Figure 3). They decreased with an increasing infestation (B1–B3), and their level increased after mite treatment (B4). A similar trend was observed in antibodies against REO, against which the hens were not immunised. On the other hand, antibodies against ORT showed an increasing trend in the B1–B3. The presence of antibodies against ORT in the blood of the hens under study, despite the absence of vaccination, indicated the occurrence of field infection. There was no correlation between mite numbers and antibody titres. Thus, there was insufficient evidence to support the hypothesis that *D. gallinae* has a negative effect on the production of post-vaccination antibodies in layer hens. Further study, including a larger sample size and negative control group, is required.

## 5. Conclusions

*D. gallinae* is currently considered to be the most bothersome ectoparasite in the commercial laying hen farms. Vaccines protect hens against pathogens, which is important from an economic perspective and consumer safety. Development of vaccine-induced immune responses might be affected by many factors. The role of *D. gallinae* as a potential immunosuppressor has been unknown. The present study was the first report in this area. The present findings suggested that *D. gallinae* might inhibit immune responses since the percentages of B cells and Th cells were negatively correlated with the number of mites. The percentage of Tc cells was positively correlated with the number of mites, which indicated that *D. gallinae* could stimulate cellular immune responses in infested laying hens. However, further research is needed to determine whether *D. gallinae* suppresses the production of vaccine-induced antibodies.

## Abbreviations

AE	avian encephalomyelitis
ANOVA	analysis of variance
B1–B4	blood collection dates correspond to the assay groups
BCR	B-cell receptor
CD	cluster of differentiation
CV%	coefficient of variation
D. gallinae	Dermanyssus gallinae
FCS	fetal calf serum
IBV	infectious bronchitis virus
IGM	immunoglobulin M
Lymp Tc	lymphocytes T cells CD3+/CD8a+ (Tc)
Lymp B	lymphocytes B IgM+ cells
Lymp Th	lymphocytes T cells CD3+/CD4+ (Th)
MANOVA	multivariate analysis of variance
MHC	major histocompatibility complex
MTI-II	mite tratment dates
NDV	Newcastle disease virus
ORT	Ornithobacterium rhinotracheale
PBS	phosphate-buffered saline
PI-II	the peak in the mite population, in this study, referred to as infestation peak
prm	poultry red mite
REO	avian reovirus
REV	reticuloendotheliosis virus
RH	relative humidity
SE	standard error
